# Optic disc and retinal vascular features in first 6 years of Chinese children

**DOI:** 10.3389/fped.2023.1101768

**Published:** 2023-03-23

**Authors:** Guina Liu, Anna Jiang, Le Cao, Saiguang Ling, Xi Wang, Shaochong Bu, Fang Lu

**Affiliations:** ^1^ Department of Ophthalmology, West China Hospital, Sichuan University, Chengdu, China; ^2^ Department of Neurology, West China Hospital, Sichuan University, Chengdu, China; ^3^ EVision Technology (Beijing) Co. LTD, Beijing, China; ^4^ Tianjin Key Laboratory of Retinal Functions and Diseases, Tianjin Branch of National Clinical Research Center for Ocular Disease, Eye Institute and School of Optometry, Tianjin Medical University Eye Hospital, Tianjin, China

**Keywords:** optic disc, retinal vasculature, children’s vision, artificial intelligence, retinal development

## Abstract

**Purpose:**

Retinal microvasculature plays an important role in children's fundus lesions and even in their later life. However, little was known on the features of normal retina in early life. The purpose of this study was to explore the normal retinal features in the first 6 years of life and provide information for future research.

**Methods:**

Children, aged from birth to 6 years old and diagnosed with various unilateral ocular diseases were included. Venous phase fundus fluorescein angiography images with the optic disc at the center were collected. Based on the ResUNet convolutional neural network, optic disc and retinal vascular features in the posterior retina were computed automatically.

**Results:**

A total of 146 normal eyes of 146 children were included. Among different age groups, no changes were shown in the optic disc diameter (y = −0.00002x + 1.362, R^2^ = 0.025, *p *= 0.058). Retinal vessel density and fractal dimension are linearly and strongly correlated (*r* = 0.979, *p *< 0.001). Older children had smaller value of fractal dimension (y = −0.000026x + 1.549, R^2 ^= 0.075, *p *= 0.001) and narrower vascular caliber if they were less than 3 years old (y = −0.008x + 84.861, R^2 ^= 0.205, *p *< 0.001). No differences were in the density (y = −0.000007x + 0.134, R^2 ^= 0.023, *p *= 0.067) and the curvature of retinal vessels (lnC = −0.00001x − 4.657, R^2 ^= 0.001, *p *= 0.667).

**Conclusions:**

Age and gender did not impact the optic disc diameter, vessel density, and vessel curvature significantly in this group of children. Trends of decreased vessel caliber in the first 3 years of life and decreased vessel complexity with age were observed. The structural characteristics provide information for future research to better understand the developmental origin of the healthy and diseased retina.

## Introduction

Retinal vessels are the only blood vessels that can be viewed directly and noninvasively in a living person with ever increasing technical sophistication (1). Previous studies pointed out that microvascular features may be important indicators for monitoring chronic systemic diseases and reflecting the conditions of infants *in utero*, which could influence the development of later life (2–5). Retinal microvasculature has also been reported to be related to hypertension and central hemodynamics in children (6, 7). The morphological features of retinal vessels in early adolescence could be valid indictors for predicting microvascular diseases in adulthood, as retinal vessel caliber was associated with body mass index and insulin resistance, which could be used as a possible noninvasive proxy for the identification of elevated risk for cerebral microvascular disease in adulthood (8). Retinal vascular geometry independently predicts the incidence of renal dysfunction in young people with type 1 diabetes (9) and the subsequent development of proliferative diabetic retinopathy (10, 11). Additionally, retinal vascular features serve as diagnostic indicators and evaluation indexes for some pediatric fundus lesions, like familial exudative vitreoretinopathy and retinopathy of prematurity (12–14). Another important structure that is often being affected in childhood fundus diseases is the optic disc, which is associated with various abnormal development of the optic nerve in children (15).

According to the Developmental Origins of Health Disease hypothesis (16), it is vital to reveal the normal development of retinal vessels in early life exposure, which could provide the baseline for studies focusing on the pathogenesis of retinal diseases. A vast body of research has focused on the characteristics of abnormal eyes to reveal the pathology of retinal vessels, especially in adult population (13, 17–19). Only several studies measured the retinal vessels in infants or young children due to the technical difficulties, cooperative issues, and ocular magnification, which are big problems in measuring pediatric eyes (20). Kandasamy et al. (21, 22) reported the retinal microvasculature measurements in full-term newborn infants and retinal microvascular development in the first 2 years. Some cohorts were established to measure the retinal microvasculature at years of 6, 11, and 6–12 (2, 23, 24). These data described the development of retinal vessels in children, at some degree. Nonetheless, due to the limitation of age range and subject ethnicities, data from these studies might not represent the development pattern of retinal vessels in Chinese children.

Currently, the data on normal retinal vascular measurement among Chinese children, especially in their early 6 years of life, are scarce. Therefore, we carried out this study to analyze the optic disc and retinal vascular parameters of the fundus fluorescein angiography (FFA) images quantitatively using ResUNet convolutional neural network (ResUNet), precisely solving problems of technical difficulties and cooperative issues in pediatric measurements. This study aimed to provide comprehensive characteristics of retinal vessels in the first 6 years of life, which would provide information for future research to better understand the developmental origin of the healthy and diseased retina.

## Materials and methods

### Study design

This cross-sectional study was performed in the department of ophthalmology, West China Hospital of Sichuan University, Chengdu, China. Children, aged from birth to 6 years old, diagnosed with various unilateral ocular diseases from 2019 to 2021 were selected. Patients with systematic diseases were excluded. Selected children were examined carefully to confirm that the contralateral eye was free of diseases and received FFA imaging under general anesthesia using the RetCam 3 system equipped with 130° wide-angle lens (Clarity Medical Systems, Inc., Pleasanton, CA, United States). Finally, the FFA images from all normal contralateral eyes of selected children were processed and measured. This study was approved by the Ethics Committee on Biomedical Research, West China Hospital of Sichuan University, and was conducted in compliance with good clinical practice guidelines, institutional review board regulations, and with written consent from parents in accordance with the tenets of the Declaration of Helsinki.

### Image processing

**Figure 1:**
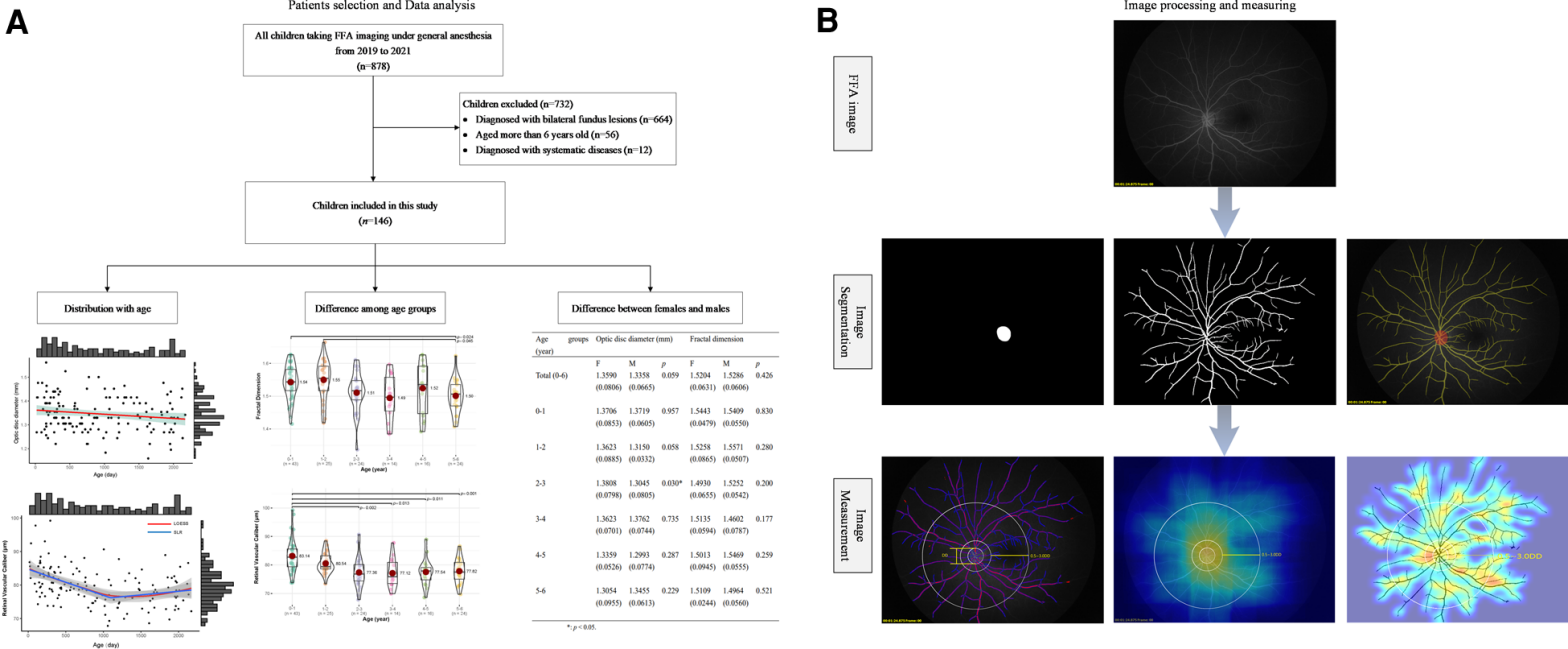
The framework of the study design. (**A**) Flowchart of recruited children from 2019 to 2021 and data analysis in this study, including the distribution of optic disc diameter and retinal vascular parameters (fractal dimension, caliber, density, and curvature of retinal vessels) with age, the difference of these indictors among several age groups, and difference of those indictors between females and males in each age group. (**B**) The presentation of image processing and measuring. Each FFA image was segmented to acquire the features of the optic disc and retinal vessels (image segmentation). Then, the optic disc diameter, fractal dimension of the whole retina, the mean caliber, density, and curvature of retinal vessels in the posterior retina were computed (image measurement). In the image measurement, the left picture showed the measurement of optic disc diameter and the caliber of retinal vessels, and each pink line represented the retinal vascular caliber at that special point; the medium picture showed the measurement of retinal vascular density, and the heat map present the value of density with the brighter color meaning the greater density; the right picture showed the measurement of retinal vascular curvature, and the heat map present the value of curvature with the brighter color meaning the greater curvature. FFA, fundus fluorescein angiography.

The venous phase FFA images with the optic disc at the center were collected for analysis. Before segmentation, the optic disc region and vascular region of FFA images were labeled using EVLabelImage program (EVision, Inc., Beijing, China) by two experienced retinal specialists. Afterward, the labeled images were sent to ResUNet for training segmentation model. The segmentation model can be obtained after the ResUNet model validation (25) (Figure 1B).

A total of 93 FFA image sets were randomly split into training, validation, and test data sets with a ratio of 7:1:2. The performance of the ResUNet validation was evaluated by accuracy, sensitivity, specificity, and intersection over union (IoU) as described previously (26, 27). Briefly, accuracy was defined as the proportion of correctly identified pixels to the entire number of pixels independent of class. Sensitivity was the proportion of correctly identified positive pixels to the entire number of positive pixels independent of class. Specificity was the proportion of correctly identified negative pixels to the entire number of negative pixels independent of class. IoU was the ratio of the number of correctly classified pixels over the number of ground truth pixels and the predicted pixels in that class. The optic disc segmentation achieved an accuracy of 0.999, with a sensitivity of 0.966, a specificity of 0.999, and an IoU of 0.940; the retinal vessel segmentation had an accuracy of 0.979, with a sensitivity of 0.857, a specificity of 0.988 and an IoU of 0.735 (Table 1).

**Table 1:** Performance of ResUNet on segmentation of optic disc and retinal vessels.

Segmentation	Accuracy	Sensitivity	Specificity	IoU
Optic disc	0.999	0.966	0.999	0.940
Retinal vessel	0.979	0.857	0.988	0.735

FN, false negative; FP, false positive; TN, true negative; TP, true positive.

Accuracy = (TP + TN)/(TP + TN + FP + TN), Sensitivity = TP/(TP + FN), Specificity = TN/(TN + FP), and IoU = TP/(TP + FP + FN).

### Image measuring

Based on previous research, the central posterior retina was defined as a circular area centered at the optic disc with a radius of three times of the optic disc diameter (DD) (28). The optic disc diameter and features of retinal vessels in this area were measured based on the segmentation model using ResUNet (Figure 1B).

The optic disk was defined by the inner border of the scleral rim surrounding the nerve tissue (29). After segmentation, the optic disc diameter was measured as the long axis of the minimum outer rectangle of the optic disc. Fractal dimensions, indicating the whole branching pattern of the retinal vascular tree, were computed from a skeletonized line tracing by using the box-counting method (30). The mean caliber (31), density (32), and curvature (33) of retinal vessels in the posterior retina (0.5–3.0 DD) were measured without distinguishing retinal arteriole and venule. Briefly, retinal vascular caliber was measured as the inner diameter without the vascular wall. Retinal vascular density was measured as the ratio of the vessel pixel area to the total pixel area. Retinal vascular curvature was measured as the reciprocal of the radius of the outer circle.

### Unit conversion

Based on previous publications (34), RetCam 3 provided images with pixels up to 1.92 million of which the horizontal pixel was 1,600 and the vertical pixel was 1,200. The conversion relationship between the pixel of RetCam 3 instrument and the actual value was 12.2 µm/pixel. According to the conversion relationship between pixels and micrometers, the diameters of the optic disc and retinal vessels were converted from pixel units to micrometer units.

### Statistical analysis

Statistical analyses were performed using R programming language version 4.2.0 (Daniel E. Ho, Stanford, CA, United States) and SPSS version 25.0 (IBM Corp., Armonk, NY, United States). The normality of variables was determined using the D’Agostino-Pearson test. Data are reported as mean ± standard deviation (SD) for data with normal distribution or median [interquartile range (IQR)] for data with skewed distribution. Linear regression was used to display the variation trend of the parameters of retinal vessels with the independent variable of age (day, D). One-way ANOVA test was used to compare the differences among all parameters among various age groups. An independent-sample t-test was used to compare the differences among all parameters in females and males. A *p*-value <0.05 is considered significant.

## Results

A total of 146 children diagnosed with various unilateral ocular diseases were included in this study with 64 (43.84%) females and 86 (58.90%) left eyes (Figure 1A and Table 2). The diagnosis of unilateral ocular diseases included monocular persistent hyperplastic primary vitreous, monocular cataract, monocular morning glory syndrome, and unilateral coats disease. The clinical characteristics are listed in Table 2. The average gestational age (GA) was 38.60 ± 2.02 (26.00–41.28) weeks. Most children (133, 91.10%) were born at term in this study, while 11 children were born prematurely and only 1 child was born with unclear gestational age. The average birth weight (BW) was 3,250 ± 586 (930–4,600) g. A total of 128 (87.67%) children had normal birth weight, 11 (7.53%) had low birth weight, and 7 (4.79%) had high birth weight. Of the included children, 97.95% were naturally conceived. Seventy-four (50.68%) children were delivered naturally and 72 (49.32%) by cesarean section. Additionally, 133 (91.10%) children in this study had no history of oxygen inhalation after their birth, while 13 (8.90%) had history of oxygen inhalation.

**Table 2:** the clinical characteristics of included children (*n* = 146).

Clinical characteristics	Number	Percentage
**Gender**		
Female	64	43.84
Male	82	56.16
**Eye**		
Left	86	58.90
Right	60	41.10
**Gestational age**		
Pre-term (GA < 37 weeks)	11	7.53
Full-term (37 weeks ≤ GA < 42 weeks)	133	91.10
Post-term (GA ≥ 42 weeks)	1	0.68
Unknown	1	0.68
**Birth weight**		
Low (BW < 2,500 g)	11	7.53
Normal (2,500 g ≤ BW ≤ 4,000 g)	128	87.67
High (BW > 4,000 g)	7	4.79
**Conception**		
Natural	143	97.95
Non-natural	3	2.05
**Parturition**		
Natural	74	50.68
Cesarean	72	49.32
**History of oxygen after birth**		
Yes	13	8.90
No	133	91.10
**Age (years)**		
0–1	43	29.45
1–2	25	17.12
2–3	24	16.44
3–4	14	9.59
4–5	16	10.96
5–6	24	16.44

GA, gestational age; BW, birth weight.

**Figure 2:**
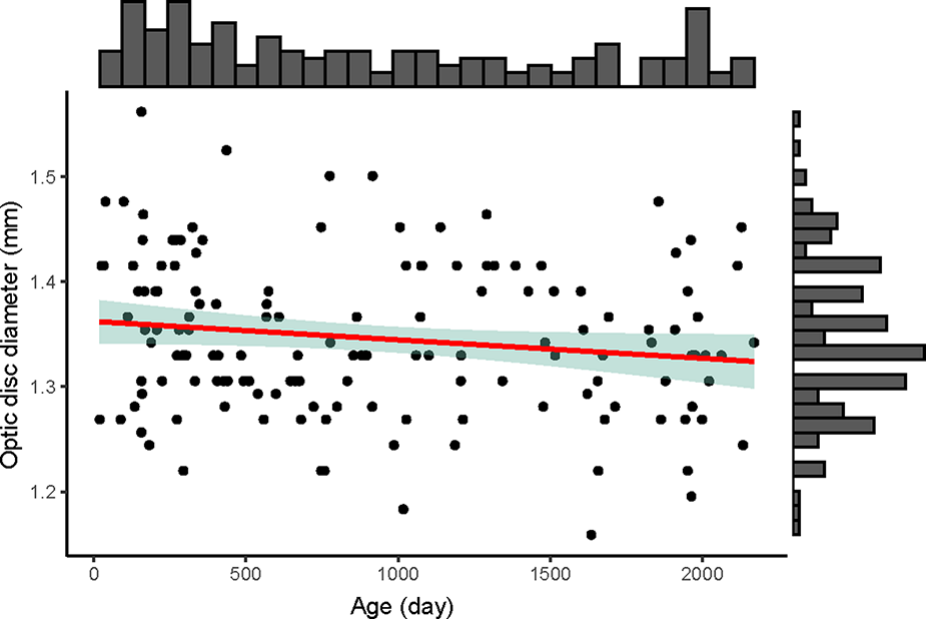
The distribution of optic disc diameter (mm) with age (day) of the 146 included children. The upper histogram showed the distribution of the age (day) and the right histogram showed the distribution of the optic disc diameter (mm). The red line was the linear regression line (y = −0.00002x + 1.362), showing no changes of optic disc diameter among different age. The green shadow area was the 95% confidence interval of the linear regression line.

Optic disc diameters were similar among different age (y = −0.00002x + 1.362, R^2^ = 0.025, *p* = 0.058) (Figure 2). The optic disc diameter of all included children was 1.35 ± 0.07 mm, 1.37 ± 0.07 mm in children aged 0–1 year old, 1.33 ± 0.05 mm in 1–2 years, 1.34 ± 0.09 mm in 2–3 years, 1.37 ± 0.07 mm in 3–4 years, 1.28 ± 0.07 mm in 4–5 years, and 1.33 ± 0.07 mm in 5–6 years (Supplementary Table S1). When compared between females and males in each age group, females had bigger optic disc in the 2–3 years group (1.38 vs. 1.30, *p* = 0.030) and no significant difference was found in other groups (Supplementary Table S2).

**Figure 3:**
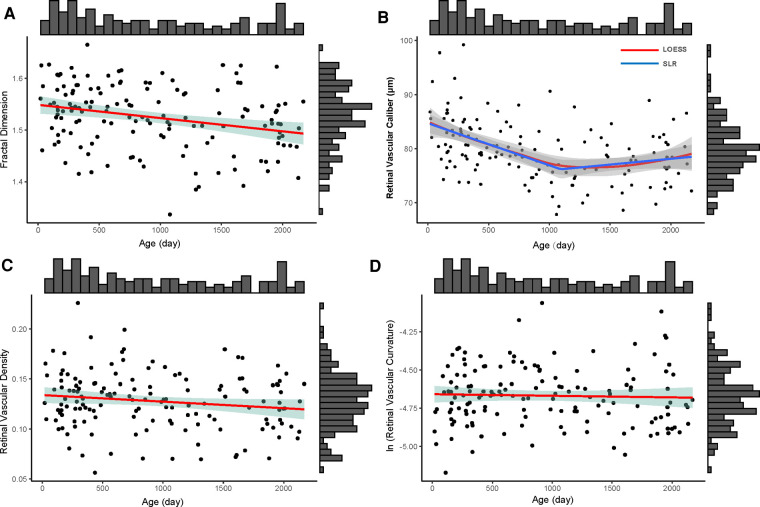
The distribution of four retinal vascular parameters with age (day). the upper histogram showed the distribution of the age of 146 included children and the right one showed the distribution of fractal dimension (**A**), retinal vascular caliber (**B**), retinal vascular density (**C**), and retinal vascular curvature (**D**) of 146 included children. (**A**) The distribution of fractal dimension of whole retinal vessels with age; the red line was the linear regression line (y = −0.000026x + 1.549); the green shadow area was the 95% confidence interval of the linear regression. (**B**) The distribution of retinal vascular caliber with age; the red line was the locally weighted regression (LOESS) line and the blue line was the segmented linear regression line (SLR, y = −0.008x + 84.861, if x < 1,095; y = 0.0001x + 6.148, if x ≥ 1,095); the gray shadow areas around LOESS line and SLR line were their 95% confidence intervals, respectively. (**C**) The distribution of retinal vascular density with age; the red line was the linear regression line (y = −0.000007x + 0.134); the green shadow area was the 95% confidence interval of the linear regression line. (**D**) The distribution of ln (retinal vascular curvature) (lnC) with age. The red line was the linear regression line (lnC = −0.00001x − 4.657); the green shadow area was the 95% confidence interval of the linear regression line.

Using correlation analysis, vessel density measurement and fractal dimension are linearly and strongly correlated (*r* = 0.979, R^2 ^= 0.958, *p *< 0.001). For fractal dimension, older children had less complex retinal vascular structures (y = −0.000026x + 1.549, R^2 ^= 0.075, *p *= 0.001) (Figure 3A). For vascular caliber, older children had narrower retinal vessels when they were less than 3 years old (y = −0.008x + 84.861, R^2 ^= 0.205, *p *< 0.001), but there were no changes in these children aged from 3 to 6 years old (y = 0.0001x + 6.148, R^2 ^= 0.011, *p *= 0.460 > 0.05) (Figure 3B). In this study, age had no impact on retinal vascular density (y = −0.000007x + 0.134, R^2 ^= 0.023, *p* = 0.067 > 0.05) and curvature of retinal vessels (lnC = −0.00001x − 4.657, R^2 ^= 0.001, *p* = 0.667) (Figures 3C,D).

**Figure 4:**
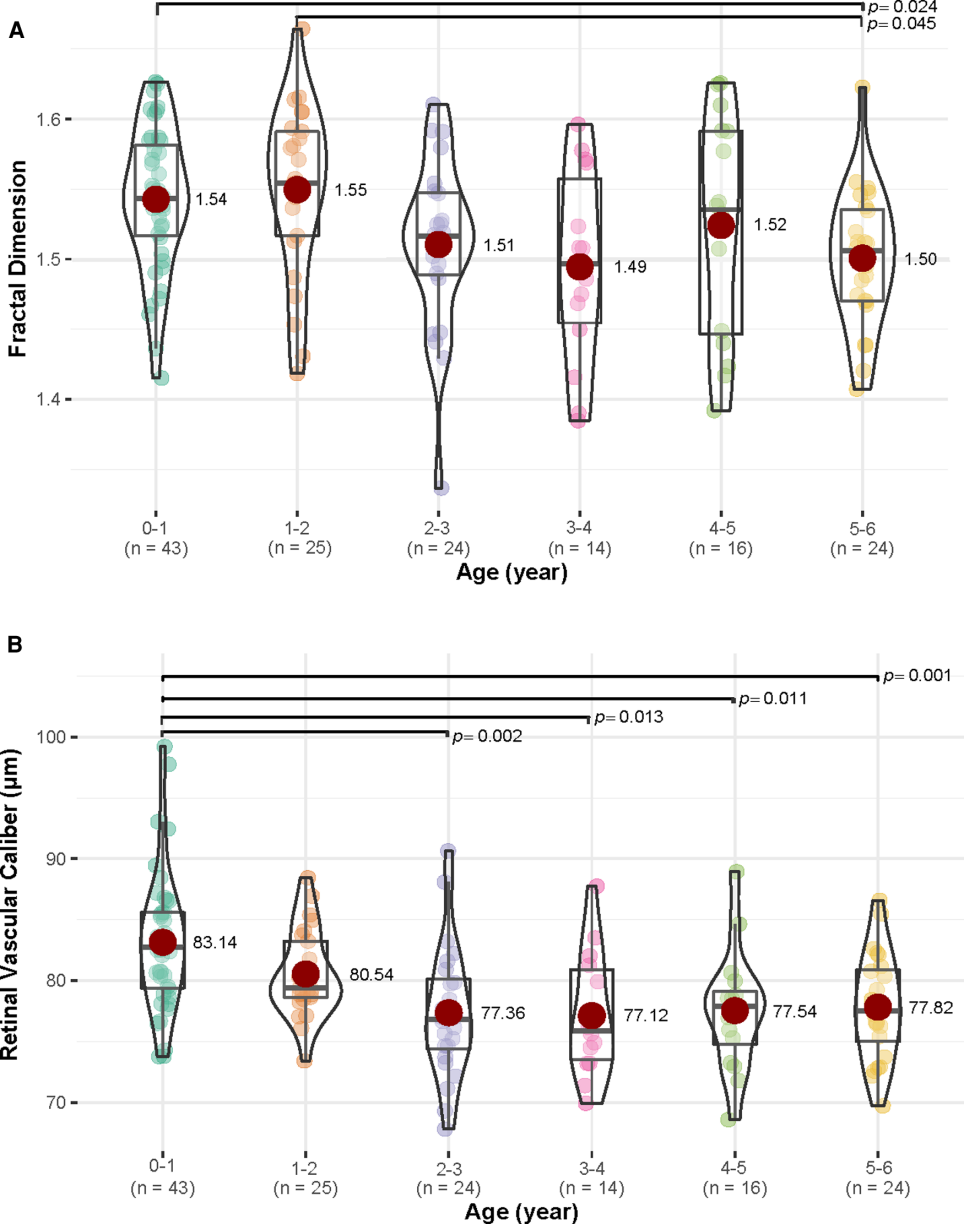
The differences in fractal dimension and retinal vascular caliber (µm) among various age groups (year). (**A**) For fractal dimension, children in 0–1 and 1–2 years age groups had more complex retinal vascular structures than those in age group 5–6 years age group (*p *< 0.05). (**B**) For retinal vascular caliber, children in age group 0–1 year had higher retinal vascular caliber than those in 2–3, 3–4, 4–5, and 5–6 years age groups (*p *< 0.05).

When compared among various age groups, children aged 0–1 year and 1–2 years had more complex retinal vascular structures than those aged 5–6 years, with *p *= 0.024 and *p *= 0.045, respectively. Children aged 0–1 year had higher retinal vascular caliber than those aged 2–3 years (*p *= 0.002), 3–4 years (*p *= 0.013), 4–5 years (*p *= 0.011), and 5–6 years (*p *= 0.001) (Figure 4). However, retinal vascular density and retinal vascular curvature showed no significant differences among different age groups (*p *> 0.05, Supplementary Table S1). When compared between females and males in each age group, females had a higher value of retinal vascular caliber than males in the 3–4 years group (79.51 vs. 72.81, *p *= 0.011, Supplementary Table S2).

## Discussion

This cross-sectional study described the retinal features measuring on the contralateral eye of the Chinese children up to 6 years old with unilateral ocular disorders based on FFA images. Our data on optic disc and retinal vessels provide information for future research to better understand the developmental origin of the healthy and diseased retina. Older children had less complex retinal vascular morphology in this study, which was in agreement with the inverse correlation of fractal dimension with age reported in previous studies (24, 35, 36). A previous study on the retinal vasculature of the Hong Kong children aged 6–8 years had a fractal dimension of about 1.24 (37). Similar study on Malay children aged 6–12 years had a fractal dimension of about 1.22 (38) and the study on 6–12-year-old Malay girls found a value of 1.42 (24). Compared with these studies, the current study had a bigger fractal dimension of all included children (1.52) and girls (1.53). The possible reason for it was that the data in this study were from FFA images, which could detect smaller vessels in the retinal microvasculature leading to a higher value of fractal dimension. Additionally, children included in the study were aged from birth to 6 years, younger than those in previous studies, which was consistent with the trend of fractal dimension. In this study, there was no difference in fractal dimension between females and males. The measurements of fractal dimension in females and males were inconsistently reported in the previous research. The research reported a bigger fractal dimension in Middle Eastern females (39), while studies on Australian or Indian population had inverse results (40, 41). The inconsistent results might come from sample size, age range, sex ratio, and ethnicity.

In this study, the retinal vessel density and fractal dimension were found to be linearly correlated. The same correlation was revealed in a previous study with the age of the study population ranging from 10 to 73 years (32). Fractal dimension is an indicator describing the morphological complexity of retinal microvasculature, but the physiological connection with diseases has not yet been clarified due to the inconsistency of image acquisition and analysis techniques (30, 32, 42). Vascular density was a quantitative index with definite physical meaning of the percentage area occupied by flowing blood vessels in the selected region, which has been used as a value could reflect the structure of vasculature in certain retinal vascular conditions (43). Therefore, our finding suggested that the vessel density measurement may be valuable for monitoring the changes of retinal microvasculature since they were not influenced by age and gender. While the correlation between the fractal dimension and vessel density and its clinical relevance may need further exploration.

Older children had a smaller value of retinal vascular caliber when less than 3 years, but it did not indicate the narrowing growth of retinal vessels in the first 3 years of life, as it has been reported that the eye still grows while it is already disproportionally large at birth in the human (44). One possible explanation for this phenomenon was that the hypermetropic state of the eye or the length of eye axis with age may have an impact on the measurement of caliber (24) resulting in the false reduction of the measurement (22). The retinal vascular caliber was unchanged if children were older than 3 years, which was consistent with these studies supporting that there was no association between age and retinal vascular caliber after correcting for ocular magnification (3, 45). Therefore, the stable retinal vascular caliber in children older than 3 years would help diagnose cardiovascular disease (46, 47), hypertension (41), and cerebrovascular diseases (48) for they would influence retinal vascular caliber. Compared with previous studies (2, 21, 22, 49, 50) computing central retinal arteriolar equivalent (about 145 µm) or central retinal venular equivalent (about 225 µm) in 0.5–1.0 DD with color fundus images, this study reported smaller average retinal vascular caliber (79.68 ± 5.47 µm) in posterior retina based on FFA images. It is possible that FFA images provided much more information on retinal microvasculature than color fundus images and more visible retinal vessels in the posterior retina were included in the calculation. For gender, females had higher value of retinal vascular caliber in the 3–4 years group, but other groups had no such difference, which was consistent with previous studies (22, 50). Due to the unbalanced sex ratio in the 3–4 years group, further analysis with larger sample size is needed in future explorations.

Consistent with previous research (51), the current study showed a stable curvature of retinal vessels without the influence of age, which is consistent with previous study (52). Therefore, the curvature of retinal vessels could be used as a referenced parameter for some vascular diseases, such as retinopathy of prematurity, hypertension, and cerebrovascular diseases (41, 48, 53). It can also provide a possible correlation between conditions *in utero* or early life and adult life (54). Once the curvature of retinal vessels changed, pathological conditions may emerge and more attention on disease monitoring would be needed.

In the current study, the diameter of the optic disc remained stable in all age groups, which was consistent with previous studies showing that the optic disc diameter did not vary significantly by age (55, 56) or whether the baby was born prematurely or as a full-term baby (34, 57). However, reports showed that the optic disc keeps growing postnatally by adding more myelin layers for 6–8 years (44). This evidence indicated little influence of age on optic disc diameter, and it would be helpful in diseases like optic neuropathy (58–60) and ischemic brain injury (61). The average of optic disc diameter in this study was 1.35 ± 0.07 mm, which was in agreement with the studies measuring optic disc size in pre-term and full-term infants using color images taken by the RetCam system (1.41 ± 0.19 mm and 1.42 ± 0.11 mm, respectively) (29, 34, 62). Females had slightly bigger optic discs than males when 2–3 years old, while previous studies reported no difference between females and males (57, 63). The possible explanation for it was the different races, limited population, and various sex ratios in each age group.

There were several limitations in this study. First, we did not consider the influence of the actual development of refractive state and the axial length, which might impact on the accuracy of absolute measurement. However, the results could be of value in each age group since most parameters like density (32), curvature (64), and fractal dimension (35) computed in this study were relative values, which were not influenced by magnification and bias produced by photographic process. Second, although we exhausted the effort to confirm diagnosis of unilateral disease in this cohort, it is possible that the studied eyes may still suffer from the underlying preexisting conditions. Therefore, the results need to be interpreted with caution. Third, an overall limited number of cases with a relevant spread in age range may lead to asymmetrical distribution of the subjects in each age group resulting in diminished power of the study. Significant differences in the diameter of the disc and the vascular density may be revealed with a larger study population in the future research. Finally, the measurement of retinal features was based on FFA images under general anesthesia, which was not suitable for comparing with other studies using color fundus images or images taken in the outpatient clinic without sedation.

In conclusion, the contralateral normal eye of the children with unilateral ocular diseases from birth to 6 years old in our study showed no significant changes in optic disc diameter, retinal vessel curvature, and density, while the vessel density and fractal dimension were correlated linearly. A trend of decreased retinal vessels caliber was shown in the first 3 years of life and kept unchanged in the later life.

## Data Availability

The original contributions presented in the study are included in the article/Supplementary Material, further inquiries can be directed to the corresponding authors.

## References

[1] Selvam S Kumar T Fruttiger M. Retinal vasculature development in health and disease. Prog Retin Eye Res. (2018) 63:1–19. 10.1016/j.preteyeres.2017.11.001 29129724

[2] Wei FF Raaijmakers A Zhang ZY van Tienoven TP Huang QF Yang WY Association between cognition and the retinal microvasculature in 11-year old children born preterm or at term. Early Hum Dev. (2018) 118:1–7. 10.1016/j.earlhumdev.2018.01.018 29413869 PMC5885985

[3] Gishti O Jaddoe VW Duijts L Steegers E Reiss I Hofman A Impact of birth parameters and early life growth patterns on retinal microvascular structure in children: the generation R study. J Hypertens. (2015) 33(7):1429–37. 10.1097/HJH.0000000000000561 25799210

[4] Gopinath B Baur LA Wang JJ Teber E Liew G Cheung N Smaller birth size is associated with narrower retinal arterioles in early adolescence. Microcirculation. (2010) 17(8):660–8. 10.1111/j.1549-8719.2010.00062.x 21044220

[5] Shariflou S Georgevsky D Mansour H Rezaeian M Hosseini N Gani F Diagnostic and prognostic potential of retinal biomarkers in early on-set Alzheimer’s disease. Curr Alzheimer Res. (2017) 14(9):1000–7. 10.2174/1567205014666170329114445 28356048

[6] Wei FF Raaijmakers A Melgarejo JD Cauwenberghs N Thijs L Zhang ZY Retinal and renal microvasculature in relation to central hemodynamics in 11-year-old children born preterm or at term. J Am Heart Assoc. (2020) 9(15):e014305. 10.1161/JAHA.119.014305 32750311 PMC7792278

[7] Yang Z Huang Y Qin Y Pang Y. Clinical characteristics and factors associated with hypertension in 205 hospitalized children: a single-center study in southwest China. Front Pediatr. (2021) 9:620158. 10.3389/fped.2021.620158 33898356 PMC8058176

[8] Tirsi A Duong M Tsui W Lee C Convit A Retinal vessel abnormalities as a possible biomarker of brain volume loss in obese adolescents. Obesity (Silver Spring). (2013) 21(12):E577–85. 10.1002/oby.20450 23512847 PMC3695074

[9] Benitez-Aguirre PZ Sasongko MB Craig ME Jenkins AJ Cusumano J Cheung N Retinal vascular geometry predicts incident renal dysfunction in young people with type 1 diabetes. Diabetes Care. (2012) 35(3):599–604. 10.2337/dc11-1177 22250064 PMC3322713

[10] Habib MS Al-Diri B Hunter A Steel DH. The association between retinal vascular geometry changes and diabetic retinopathy and their role in prediction of progression–an exploratory study. BMC Ophthalmol. (2014) 14:89. 10.1186/1471-2415-14-89 25001248 PMC4094636

[11] Peña AS Liew G Anderson J Giles LC Gent R Wong TY Early atherosclerosis is associated with retinal microvascular changes in adolescents with type 1 diabetes. Pediatr Diabetes. (2018) 19(8):1467–70. 10.1111/pedi.12764 30175493

[12] Gilmour DF. Familial exudative vitreoretinopathy and related retinopathies. Eye (Lond). (2015) 29(1):1–14. 10.1038/eye.2014.70 25323851 PMC4289842

[13] Hartnett ME Penn JS. Mechanisms and management of retinopathy of prematurity. N Engl J Med. (2012) 367(26):2515–26. 10.1056/NEJMra1208129 23268666 PMC3695731

[14] Chaves-Samaniego MJ García Castejón M Chaves-Samaniego MC Solans Perez Larraya A Ortega Molina JM Muñoz Hoyos A Risk calculator for retinopathy of prematurity requiring treatment. Front Pediatr. (2020) 8:529639. 10.3389/fped.2020.529639 33042928 PMC7530187

[15] Jeng-Miller KW Cestari DM Gaier ED. Congenital anomalies of the optic disc: insights from optical coherence tomography imaging. Curr Opin Ophthalmol. (2017) 28(6):579–86. 10.1097/ICU.0000000000000425 28817389

[16] Hanson MA Gluckman PD. Early developmental conditioning of later health and disease: physiology or pathophysiology? Physiol Rev. (2014) 94(4):1027–76. 10.1152/physrev.00029.2013 25287859 PMC4187033

[17] Konstantinidis L Guex-Crosier Y. Hypertension and the eye. Curr Opin Ophthalmol. (2016) 27(6):514–21. 10.1097/ICU.0000000000000307 27662019

[18] Nguyen TT Wang JJ Wong TY. Retinal vascular changes in pre-diabetes and prehypertension: new findings and their research and clinical implications. Diabetes Care. (2007) 30(10):2708–15. 10.2337/dc07-0732 17595350

[19] Forster RB Garcia ES Sluiman AJ Grecian SM McLachlan S MacGillivray TJ Retinal venular tortuosity and fractal dimension predict incident retinopathy in adults with type 2 diabetes: the Edinburgh type 2 diabetes study. Diabetologia. (2021) 64(5):1103–12. 10.1007/s00125-021-05388-5 33515071 PMC8012328

[20] Nguyen TP Ni S Khan S Wei X Ostmo S Chiang MF Advantages of widefield optical coherence tomography in the diagnosis of retinopathy of prematurity. Front Pediatr. (2021) 9:797684. 10.3389/fped.2021.797684 35118032 PMC8806029

[21] Kandasamy Y Smith R Wright IM. Retinal microvascular development in the first two years. Microvasc Res. (2019) 125:103875. 10.1016/j.mvr.2019.04.005 30981745

[22] Kandasamy Y Smith R Wright IM. Retinal microvasculature measurements in full-term newborn infants. Microvasc Res. (2011) 82(3):381–4. 10.1016/j.mvr.2011.07.011 21840326

[23] Gopinath B Baur LA Wang JJ Hardy LL Teber E Kifley A Influence of physical activity and screen time on the retinal microvasculature in young children. Arterioscler Thromb Vasc Biol. (2011) 31(5):1233–9. 10.1161/ATVBAHA.110.219451 21508347

[24] Tai EL Li LJ Wan-Hazabbah WH Wong TY Shatriah I. Effect of axial eye length on retinal vessel parameters in 6 to 12-year-old Malay girls. PLoS One. (2017) 12(1):e0170014. 10.1371/journal.pone.0170014 28107389 PMC5249240

[25] Zhang ZX Liu QJ Wang YH. Road extraction by deep residual U-net. IEEE Geosci Remote Sens Lett. (2018) 15(5):749–53. 10.1109/LGRS.2018.2802944

[26] Adamidi ES Mitsis K Nikita KS. Artificial intelligence in clinical care amidst COVID-19 pandemic: a systematic review. Comput Struct Biotechnol J. (2021) 19:2833–50. 10.1016/j.csbj.2021.05.010 34025952 PMC8123783

[27] Alshayeji MH ChandraBhasi Sindhu S Abed S. CAD systems for COVID-19 diagnosis and disease stage classification by segmentation of infected regions from CT images. BMC Bioinformatics. (2022) 23(1):264. 10.1186/s12859-022-04818-4 35794537 PMC9261058

[28] Brown JM Campbell JP Beers A Chang K Ostmo S Chan RVP Automated diagnosis of plus disease in retinopathy of prematurity using deep convolutional neural networks. JAMA Ophthalmol. (2018) 136(7):803–10. 10.1001/jamaophthalmol.2018.1934 29801159 PMC6136045

[29] De Silva DJ Cocker KD Lau G Clay ST Fielder AR Moseley MJ. Optic disk size and optic disk-to-fovea distance in preterm and full-term infants. Invest Ophthalmol Vis Sci. (2006) 47(11):4683–6. 10.1167/iovs.06-0152 17065474

[30] Nadal J Deverdun J de Champfleur NM Carriere I Creuzot-Garcher C Delcourt C Retinal vascular fractal dimension and cerebral blood flow, a pilot study. Acta Ophthalmol. (2020) 98(1):e63–71. 10.1111/aos.14232 31545560

[31] Cheung CY Ong S Ikram MK Ong YT Chen CP Venketasubramanian N Retinal vascular fractal dimension is associated with cognitive dysfunction. J Stroke Cerebrovasc Dis. (2014) 23(1):43–50. 10.1016/j.jstrokecerebrovasdis.2012.09.002 23099042

[32] Ab Hamid F Che Azemin MZ Salam A Aminuddin A Mohd Daud N Zahari I. Retinal vasculature fractal dimension measures vessel density. Curr Eye Res. (2016) 41(6):823–31. 10.3109/02713683.2015.1056375 26268475

[33] Tan P Lye DC Yeo TK Cheung CY Thein TL Wong JG A prospective case-control study to investigate retinal microvascular changes in acute dengue infection. Sci Rep. (2015) 5:17183. 10.1038/srep17183 26603217 PMC4658599

[34] Feng X Nan Y Pan J Zou R Shen L Chen F. Comparative study on optic disc features of premature infants and full-term newborns. BMC Ophthalmol. (2021) 21(1):120. 10.1186/s12886-021-01833-6 33676441 PMC7936456

[35] Liew G Wang JJ Cheung N Zhang YP Hsu W Lee ML The retinal vasculature as a fractal: methodology, reliability, and relationship to blood pressure. Ophthalmology. (2008) 115(11):1951–6. 10.1016/j.ophtha.2008.05.029 18692247

[36] Azemin MZ Kumar DK Wong TY Wang JJ Mitchell P Kawasaki R Age-related rarefaction in the fractal dimension of retinal vessel. Neurobiol Aging. (2012) 33(1):194.e1–4. 10.1016/j.neurobiolaging.2010.04.010 20472327

[37] Ho A Cheung CY Wong JS Zhang Y Tang FY Kam KW Independent and synergistic effects of high blood pressure and obesity on retinal vasculature in young children: the Hong Kong children eye study. J Am Heart Assoc. (2021) 10(3):e018485. 10.1161/JAHA.120.018485 33496185 PMC7955451

[38] Tai ELM Kueh YC Wan Hitam WH Wong TY Shatriah I. Comparison of retinal vascular geometry in obese and non-obese children. PLoS One. (2018) 13(2):e0191434. 10.1371/journal.pone.0191434 29389952 PMC5794084

[39] Van Craenendonck T Gerrits N Buelens B Petropoulos IN Shuaib A Standaert A Retinal microvascular complexity comparing mono- and multifractal dimensions in relation to cardiometabolic risk factors in a Middle Eastern population. Acta Ophthalmol. (2021) 99(3):e368–77. 10.1111/aos.14598 32940010

[40] Aliahmad B Kumar DK Sarossy MG Jain R. Relationship between diabetes and grayscale fractal dimensions of retinal vasculature in the Indian population. BMC Ophthalmol. (2014) 14:152. 10.1186/1471-2415-14-152 25434291 PMC4265429

[41] Wang SB Mitchell P Liew G Wong TY Phan K Thiagalingam A A spectrum of retinal vasculature measures and coronary artery disease. Atherosclerosis. (2018) 268:215–24. 10.1016/j.atherosclerosis.2017.10.008 29050745

[42] Che Azemin MZ Ab Hamid F Aminuddin A Wang JJ Kawasaki R Kumar DK. Age-related rarefaction in retinal vasculature is not linear. Exp Eye Res. (2013) 116:355–8. 10.1016/j.exer.2013.10.010 24512773

[43] Vo Kim S Semoun O Pedinielli A Jung C Miere A Souied EH. Optical coherence tomography angiography quantitative assessment of exercise-induced variations in retinal vascular plexa of healthy subjects. Invest Ophthalmol Vis Sci. (2019) 60(5):1412–9. 10.1167/iovs.18-24389 30943289

[44] Van Cruchten S Vrolyk V Perron Lepage MF Baudon M Voute H Schoofs S Pre- and postnatal development of the eye: a Species comparison. Birth Defects Res. (2017) 109(19):1540–67. 10.1002/bdr2.1100 28941218

[45] Cheung N Tikellis G Saw SM Amirul Islam FM Mitchell P Wang JJ Relationship of axial length and retinal vascular caliber in children. Am J Ophthalmol. (2007) 144(5):658–62. 10.1016/j.ajo.2007.07.023 17869206

[46] Lona G Endes K Köchli S Infanger D Zahner L Hanssen H. Retinal vessel diameters and blood pressure progression in children. Hypertension. (2020) 76(2):450–7. 10.1161/HYPERTENSIONAHA.120.14695 32594800

[47] He Y Li SM Zhang Q Cao K Kang MT Liu LR The performance of an integrated model including retinal information in predicting childhood hypertension. Pediatr Res. (2022) 91(6):1600–5. 10.1038/s41390-021-01535-1 33947999

[48] Rim TH Teo AWJ Yang HHS Cheung CY Wong TY. Retinal vascular signs and cerebrovascular diseases. J Neuroophthalmol. (2020) 40(1):44–59. 10.1097/WNO.0000000000000888 31977663

[49] Cheung N Islam FM Saw SM Shankar A de Haseth K Mitchell P Distribution and associations of retinal vascular caliber with ethnicity, gender, and birth parameters in young children. Invest Ophthalmol Vis Sci. (2007) 48(3):1018–24. 10.1167/iovs.06-0978 17325141

[50] He Y Li SM Kang MT Liu LR Li H Wei SF Association between blood pressure and retinal arteriolar and venular diameters in Chinese early adolescent children, and whether the association has gender difference: a cross-sectional study. BMC Ophthalmol. (2018) 18(1):133. 10.1186/s12886-018-0799-x 29866094 PMC5987453

[51] Byrne MP McMillan KR Coats B. Morphological analysis of retinal microvasculature to improve understanding of retinal hemorrhage mechanics in infants. Invest Ophthalmol Vis Sci. (2020) 61(3):16. 10.1167/iovs.61.3.16 32176264 PMC7401705

[52] Owen CG Rudnicka AR Nightingale CM Mullen R Barman SA Sattar N Retinal arteriolar tortuosity and cardiovascular risk factors in a multi-ethnic population study of 10-year-old children; the Child Heart and Health Study in England (CHASE). Arterioscler Thromb Vasc Biol. (2011) 31(8):1933–8. 10.1161/ATVBAHA.111.225219 21659645 PMC3145146

[53] Ghergherehchi L Kim SJ Campbell JP Ostmo S Chan RVP Chiang MF. Plus disease in retinopathy of prematurity: more than meets the ICROP? Asia Pac J Ophthalmol (Phila). (2018) 7(3):152–5. 10.22608/APO.201863 29797825 PMC7880619

[54] Li LJ Du R Loy SL Chong YS Chan JKY Wong TY Retinal microvasculature and risk of spontaneous abortion in multiethnic Southeast Asian women. Fertil Steril. (2022) 118(4):748–57. 10.1016/j.fertnstert.2022.06.033 35981917

[55] Drobnjak D Taarnhøj NC Mitchell P Wang JJ Tan A Kessel L Heritability of optic disc diameters: a twin study. Acta Ophthalmol. (2011) 89(2):e193–8. 10.1111/j.1755-3768.2010.01923.x 20636443

[56] Mansour AM. Racial variation of optic disc size. Ophthalmic Res. (1991) 23(2):67–72. 10.1159/000267091 1870843

[57] Kandasamy Y Smith R Wright IM Hartley L. Optic disc measurements in full term infants. Br J Ophthalmol. (2012) 96(5):662–4. 10.1136/bjophthalmol-2011-300950 22286069

[58] Alvarez E Wakakura M Khan Z Dutton GN. The disc-macula distance to disc diameter ratio: a new test for confirming optic nerve hypoplasia in young children. J Pediatr Ophthalmol Strabismus. (1988) 25(3):151–4. 10.3928/0191-3913-19880501-11 3397860

[59] Kelly JP Baran F Phillips JO Weiss AH. Optical coherence tomography in optic nerve hypoplasia: correlation with optic disc diameter, nerve fiber layer thickness, and visual function. J Neuroophthalmol. (2018) 38(3):312–9. 10.1097/WNO.0000000000000596 29252690

[60] Ramos Cdo V Bellusci C Savini G Carbonelli M Berezovsky A Tamaki C Association of optic disc size with development and prognosis of Leber’s hereditary optic neuropathy. Invest Ophthalmol Vis Sci. (2009) 50(4):1666–74. 10.1167/iovs.08-2695 19098324

[61] McLoone E O'Keefe M Donoghue V McLoone S Horgan N Lanigan B. Retcam image analysis of optic disc morphology in premature infants and its relation to ischaemic brain injury. Br J Ophthalmol. (2006) 90(4):465–71. 10.1136/bjo.2005.078519 16547329 PMC1856984

[62] Huynh SC Wang XY Rochtchina E Crowston JG Mitchell P. Distribution of optic disc parameters measured by OCT: findings from a population-based study of 6-year-old Australian children. Invest Ophthalmol Vis Sci. (2006) 47(8):3276–85. 10.1167/iovs.06-0072 16877392

[63] Hellström A Svensson E. Optic disc size and retinal vessel characteristics in healthy children. Acta Ophthalmol Scand. (1998) 76(3):260–7. 10.1034/j.1600-0420.1998.760302.x 9686834

[64] Lotmar W Freiburghaus A Bracher D. Measurement of vessel tortuosity on fundus photographs. Albrecht Von Graefes Arch Klin Exp Ophthalmol. (1979) 211(1):49–57. 10.1007/BF00414653 313721

