# Supplementary material for: Optic disc and retinal vascular features in first 6 years of Chinese children
Source: Front Pediatr. 2023 Mar 23;11:1101768. doi: 10.3389/fped.2023.1101768 (PMC10077150; doi:10.3389/fped.2023.1101768)
Supplement: Supplementary file 1 [file Datasheet1.docx]

**Table S1** **Retinal parameters (mean (SD) or median [IQR]) in various age groups.**

| **Age groups (year)** | **Diameter of optic disc (mm)** | **Fractal dimension** | **Density of vessels** | **Caliber of vessels (μm)** | **Curvature of vessels** |
| --- | --- | --- | --- | --- | --- |
| Total (0-6) | 1.3459 (0.0737) | 1.5250 (0.0616) | 0.1280 (0.0286) | 79.6794 (5.4656) | 0.0094 [0.0083, 0.0107] |
| 0-1 | 1.3713 (0.0739) | 1.5427 (0.0507) | 0.1308 (0.0274) | 83.1442 (5.6218) | 0.0090 [0.0081, 0.0107] |
| 1-2 | 1.3264 (0.0537) | 1.5496 (0.0606) | 0.1393 (0.0334) | 80.5400 (3.5307) | 0.0097 [0.0093, 0.0114] |
| 2-3 | 1.3394 (0.0875) | 1.5104 (0.0606) | 0.1230 (0.0251) | 77.3602 (5.3961) | 0.0099 [0.0086, 0.0112] |
| 3-4 | 1.3673 (0.0691) | 1.4945 (0.0692) | 0.1183 (0.0253) | 77.1162 (5.0642) | 0.0089 [0.0083, 0.0099] |
| 4-5 | 1.2821 (0.0667) | 1.5241 (0.0785) | 0.1288 (0.0348) | 77.5371 (4.8995) | 0.0092 [0.0074, 0.0100] |
| 5-6 | 1.3338 (0.0731) | 1.5007 (0.0488) | 0.1211 (0.0234) | 77.8201 (4.3249) | 0.0087 [0.0077, 0.0109] |
| P | 0.045* | 0.005^†^ | 0.152 | <0.001^‡^ | 0.250^§^ |

*: *p*=0.045 < 0.05, but there was no difference among various age groups.

†: group (0-1) vs. group (5-6): *p*=0.024 < 0.05; group (1-2) vs. group (5-6): *p*=0.045 < 0.05.

‡: group (0-1) vs. group (2-3): *p*=0.002 < 0.05; group (0-1) vs. group (3-4): *p*=0.013 < 0.05; group (0-1) vs. group (4-5): *p*=0.011 < 0.05; group (0-1) vs. group (5-6): *p*=0.001 < 0.05).

§: due to the skewed distribution of curvature of vessels, the LnC was used for the one-way ANOVA test.

**Table S2 Retinal parameters (mean (SD) or median [IQR])** **in females and males among various age groups.**

| **Age groups (year)**  **[Female : Male]** | **Optic disc diameter (mm)** | | | **Fractal dimension** | | | **Density of vessels** | | | **Caliber of vessels (μm)** | | | **Curvature of vessels** | | |
| --- | --- | --- | --- | --- | --- | --- | --- | --- | --- | --- | --- | --- | --- | --- | --- |
|  | F | M | *p* | F | M | *p* | F | M | *p* | F | M | *p* | F | M | *p*^†^ |
| Total (0-6)  [64:82] | 1.3590 (0.0806) | 1.3358 (0.0665) | 0.059 | 1.5204 (0.0631) | 1.5286 (0.0606) | 0.426 | 0.1268 (0.0293) | 0.1288 (0.0281) | 0.670 | 79.9763 (5.8157) | 79.4486 (5.2021) | 0.564 | 0.0090 [0.0079, 0.0110] | 0.0094 [0.0085, 0.0106] | 0.339 |
| 0-1  [23:20] | 1.3706 (0.0853) | 1.3719 (0.0605) | 0.957 | 1.5443 (0.0479) | 1.5409 (0.0550) | 0.830 | 0.1345 (0.0292) | 0.1266 (0.0252) | 0.350 | 83.4597 (5.6583) | 82.7822 (5.7048) | 0.698 | 0.0089 [0.0077, 0.0107] | 0.0096 [0.0084, 0.0106] | 0.315 |
| 1-2  [6:19] | 1.3623 (0.0885) | 1.3150 (0.0332) | 0.058 | 1.5258 (0.0865) | 1.5571 (0.0507) | 0.280 | 0.1237 (0.0454) | 0.1442 (0.0284) | 0.196 | 78.6940 (3.0675) | 81.1218 (3.5368) | 0.145 | 0.0099 [0.0080, 0.0130] | 0.0097 [0.0094, 0.0114] | 0.818 |
| 2-3  [11:13] | 1.3808 (0.0798) | 1.3045 (0.0805) | 0.030* | 1.4930 (0.0655) | 1.5252 (0.0542) | 0.200 | 0.1153 (0.0263) | 0.1296 (0.0230) | 0.169 | 77.0280 (5.9182) | 77.6420 (5.1399) | 0.788 | 0.0099 [0.0086, 0.0111] | 0.0099 [0.0085, 0.0115] | 0.607 |
| 3-4  [9:5] | 1.3623 (0.0701) | 1.3762 (0.0744) | 0.735 | 1.5135 (0.0594) | 1.4602 (0.0787) | 0.177 | 0.1265 (0.0188) | 0.1035 (0.0307) | 0.104 | 79.5088 (4.5590) | 72.8108 (2.3960) | 0.011* | 0.0091 [0.0079, 0.0104] | 0.0087 [0.0082, 0.0096] | 0.601 |
| 4-5  [8:8] | 1.3359 (0.0526) | 1.2993 (0.0774) | 0.287 | 1.5013 (0.0945) | 1.5469 (0.0555) | 0.259 | 0.1228 (0.0407) | 0.1348 (0.0293) | 0.510 | 78.5070 (6.4106) | 76.5672 (2.8619) | 0.448 | 0.0087 [0.0073, 0.0100] | 0.0096 [0.0077, 0.0108] | 0.347 |
| 5-6  [7:17] | 1.3054 (0.0955) | 1.3455 (0.0613) | 0.229 | 1.5109 (0.0244) | 1.4964 (0.0560) | 0.521 | 0.1274 (0.0112) | 0.1185 (0.0268) | 0.412 | 76.5434 (3.8417) | 78.3460 (4.5103) | 0.365 | 0.0098 [0.0078, 0.0123] | 0.0087 [0.0076, 0.0100] | 0.438 |

*: *p* < 0.05.

†: due to the skewed distribution of curvature of vessels, the LnC was used for the independent sample t-test.
